# Identification of Age and Underlying Disease Characteristics in Patients with Mild to Moderate Depression Comorbid with Parkinson’s Disease: A Retrospective Case-control Study

**DOI:** 10.62641/aep.v53i2.1702

**Published:** 2025-03-05

**Authors:** Yiran Meng, Xiaoning Li, Wei Wang, Cixiang Dai, Wanchen Li, Junfei Li, Liyan Pan

**Affiliations:** ^1^Department of Neurology, Hebei Yanda Hospital, 065201 Langfang, Hebei, China

**Keywords:** age, disease characteristics, depression, Parkinson’s disease

## Abstract

**Background:**

Depression is a widely recognized neuropsychiatric condition that often occurs as a comorbidity with various medical illnesses, including neurodegenerative disorders like Parkinson’s disease (PD). This study aimed to identify the age of onset and underlying disease characteristics associated with patients exhibiting mild to moderate depression comorbid with PD.

**Methods:**

This retrospective case-control study included 114 elderly patients (age ≥65 years) diagnosed with Parkinson’s disease. The patients were divided into two groups: the non-depressed group (n = 65) and the mild to moderate depression group (n = 49). Patients’ emotional and affective symptoms, cognitive function, and clinical characteristics were assessed using standardized scales. Statistical analyses, including chi-square tests, Wilcoxon rank-sum tests, and logistic regression analysis, were performed to evaluate associations and correlations between the variables of interest.

**Results:**

Our findings revealed that patients in the mild to moderate depression group exhibited a significantly lower onset age of PD (52.33 ± 3.87 years) compared to the non-depressed group (59.27 ± 3.62 years, *p* < 0.001). Furthermore, patients with mild to moderate depression showed significantly higher scores in mood and affective symptoms measures, including the Hamilton Anxiety Scale (HAM-A) (*p* < 0.001) and Apathy Scale (*p* < 0.001). Additionally, the duration of Parkinson’s disease was significantly longer in the mild to moderate depression group (6.78 ± 2.01 years) compared to the non-depressed group (3.45 ± 1.52 years, *p* < 0.001). Similarly, patients in the mild to moderate depression group exhibited significantly poorer performance on the Mini-Mental State Examination (MMSE) (*p* < 0.001), Montreal Cognitive Assessment (MoCA) (*p* = 0.025), verbal fluency (*p* < 0.001), and Trail Making Test (*p* = 0.005). Additionally, correlation and logistic regression analysis revealed associations and predictive value of these variables with the presence of mild to moderate depression in Parkinson’s disease.

**Conclusion:**

The study highlights the complex interaction of age and underlying disease characteristics in patients with mild to moderate depression comorbid with Parkinson’s disease. Early recognition and tailored management of depressive symptoms, mood and affective disturbances, cognitive impairment, and disease-specific characteristics are crucial for optimizing patient care and improving outcomes in individuals with Parkinson’s disease. These findings underscore the need for a comprehensive, patient-centered approach that considers the diverse interaction of demographic, clinical, and cognitive variables.

## Introduction

Depression is a widely recognized neuropsychiatric condition that frequently occurs as a comorbidity with various medical illnesses, including neurodegenerative disorders like Parkinson’s disease (PD) [1, 2, 3]. In PD patients, the prevalence of depression ranges between 20% and 30% [4]. This considerable variability underscores the need for a comprehensive understanding of the complex interaction of various demographic, clinical, and cognitive factors contributing to depression in these patients.

Patients with PD often face a range of challenges, including motor, cognitive, and emotional disturbances, which collectively impact their overall quality of life and well-being [5, 6]. While motor symptoms have received substantial attention in PD, non-motor symptoms like depression have gained increasing recognition for their significant impact on patient outcomes [7, 8]. Depression in individuals with PD has been associated with poorer health-related quality of life, increased disability, cognitive impairment, and significant caregiver burden [9, 10]. Moreover, identifying and managing depression in PD are critical, as it is linked to elevated disease progression and a poorer prognosis.

Despite the increasing recognition of depression as a common comorbidity in PD, the complex interaction of age and disease characteristics involved in the manifestation of mild to moderate depression in these patients remains underexplored [11, 12]. To address this knowledge gap, the present retrospective case-control study aimed to comprehensively identify and evaluate the associations of demographic, clinical, and cognitive variables with mild to moderate depression in elderly patients with PD. The focus on elderly patients lies in the dynamic nature of PD, where older age is a significant risk factor for the development and progression of the disease. Furthermore, understanding the demographic and disease-specific factors associated with depression among these patients can aid in tailored risk stratification, early recognition, and personalized management approaches. This comprehensive understanding of mild to moderate depression in elderly patients holds promise for developing individualized strategies to improve the overall well-being and prognosis of individuals with PD and comorbid depression.

## Materials and Methods

### Study Participants

This retrospective study included 114 elderly patients (age ≥65 years) who sought medical attention for PD or Parkinson’s syndrome from April 2022 to December 2023. Based on the severity of depression, these patients were divided into two groups: the non-depressed group (n = 65) and the mild to moderate depression group (n = 49). Informed consent was waived by the Institutional Review Board and Ethics Committee of Hebei Yanda Hospital for this study due to the exclusive use of de-identified patient data, which posed no potential harm or impact on patient care. This study was approved by the Institutional Review Board and Ethics Committee of Hebei Yanda Hospital (ethical approval No. 2022-06-001) and performed following the declaration of Helsinki guidelines.

### Inclusion and Exclusion Criteria

The inclusion criteria were set as follows: the patients clinically diagnosed idiopathic PD [13], aged ≥65 years old, those with depression met at least two of the Diagnostic and Statistical Manual of Mental Disorders (DSM-5) criteria, and with complete clinical data.

However, exclusion criteria included patients with severe illnesses that would impede questionnaire completion, inability to understand the questionnaire content or disagree to participate, patients currently receiving pharmacologic (e.g., antidepressants) or surgical treatments (e.g., deep brain stimulation), patients with psychiatric disorders (e.g., schizophrenia, psychosis, or major depressive disorder) other than mild to moderate depression, or those currently participating in another behavioral or pharmacologic trial or scheduled exercise program, and patients with significant cognitive impairment.

### Grouping Method

Based on the severity of depression associated with PD, study participants were divided into two groups: the non-depressed group (n = 65) and the mild to moderate depression group (n = 49). Depression status was assessed using the DSM-5 criteria [14, 15], with the diagnosis confirmed by two experienced psychological experts. The DSM-5 specifier requires the presence of at least two of the five criterion symptoms for most depressive episodes. The five symptoms of the anxious distress specifier include feeling keyed up or tense, feeling restless, difficulty concentrating because of worry, fear that something awful might happen, and feeling that one might lose control.

### Observation Indicators

The observational measures included patients’ general demographic characteristics (age, onset age of PD, gender, Body Mass Index (BMI), education level, smoking history, alcohol history, diabetes, hypertension, hyperlipidemia, residence, and marital status), emotional and affective symptoms (Hamilton Anxiety Scale (HAM-A) score, Apathy Scale Score), clinical features of PD (levodopa equivalent daily dose, duration of Parkinson’s disease, motor complications, disease type, H-Y staging), and cognitive function (Mini-Mental State Examination (MMSE) score, Montreal Cognitive Assessment (MoCA) score, verbal fluency, Trail Making Test).

### Emotional and Affective Symptoms

The Hamilton Anxiety Scale and the Apathy Scale were utilized to assess the emotional and affective states of patients in the experimental groups. The assessment was conducted by two experienced mental psychological experts.

The Hamilton Anxiety Scale comprises 14 items, each scored from 0 to 4, resulting in a total score of 0–6 indicating no anxiety symptoms, 7–13 suggesting possible anxiety, 14–20 indicating definite presence of anxiety, 21–28 representing evident anxiety, and scores ≥29 signifying severe anxiety. Its Cronbach’s alpha is 0.92 [16].

The Apathy Scale consists of three subscales to assess apathy subtypes related to executive, emotional, or self-generated cognitive impairment. It includes 24 items on a 4-point scale, with total scores ranging from 0 to 72. Higher scores indicate greater severity of apathy. The Cronbach’s alpha for this scale is 0.85 [17].

### Cognitive Function Assessment

Two experienced neurology experts assessed the cognitive function of the patients using the MMSE and MoCA scales.

The MMSE is a neuropsychological test used for evaluating a patient’s intellectual status and cognitive impairment. It yields a total score of 30 points, with 30 points indicating normal cognitive function (no cognitive impairment), 27–30 points indicating mild cognitive impairment, 21–26 points indicating moderate cognitive impairment, and 0–20 points indicating severe cognitive impairment. The Cronbach alpha coefficient for MMSE is 0.71 [18].

The MoCA scale includes 11 items across 8 cognitive domains, including attention and concentration, executive functions, memory, language, visuospatial skills, abstract thinking, calculation, and orientation. It yields a total score of 30 points, with scores ≥26 indicating normal cognitive function, 21–25 indicating moderate cognitive impairment, and 0–20 indicating severe cognitive impairment. The Cronbach alpha coefficient for MoCA is 0.75 [19].

### Data Cleaning and Management

Before conducting the data analysis, we used a standardized data-cleaning process to identify and rectify any inconsistencies, errors, or missing values. This process involved careful examination of the dataset, removing duplicate entries, correcting data input errors, and handling missing values. Data processing was conducted using Datawig and Pandas libraries in Python 3.6.0 (Python Software Foundation, Amsterdam, Netherlands), utilizing deep neural networks to impute missing values. Missing data was kept within 5% to control potential selection bias, and sensitivity analysis was performed. The outcomes were separately calculated for the lost to follow-up cases based on the worst and best-case scenarios. In case of no significant difference, the impact of lost to follow-up on the outcomes was minimal, making the conclusions more reliable. The final results were reported after imputing the missing values.

### Post-hoc Analysis

Using G*Power 3.1.9.7, the “Means: Difference between two independent means (two groups)” option based on *t*-tests was selected for post hoc analysis. The settings were as follows: two-tailed mode, effect size d = 0.6, α error probability = 0.05. Subsequently, the sample sizes for the two groups were entered, and the power (1-β err prob) was calculated, resulting in 0.844.

### Statistical Analysis

The data were analyzed using SPSS 29.0 statistical software (SPSS Inc., Chicago, IL, USA). Categorical data were expressed as [n (%)]. The chi-square test was applied with the basic formula when the sample size was ≥40 and the theoretical frequency T was ≥5, with the test statistic represented by χ^2^. When the sample size was ≥40 but the theoretical frequency was 1 ≤ T < 5, the chi-square test was adjusted using the correction formula. For sample size <40 or T <1, statistical analysis was conducted using Fisher’s exact probability method.

Continuous variables were initially tested for normal distribution using the Shapiro-Wilk method. Normally distributed continuous data were expressed as ($\bar{x}$ ± s). Non-normally distributed data were analyzed using the Wilcoxon rank-sum test and presented as [median (25% quantile,75% quantile)]. A *p*-value < 0.05 was considered statistically significant.

The correlation analysis was performed using Pearson correlation analysis for continuous variables and Spearman correlation analysis for categorical variables. Variables with significant distribution in difference and correlation analyses were included as covariates in logistic regression analysis.

## Results

### Demographic Characteristics

In this retrospective case-control study, we compared the demographic characteristics of mild to moderate depression patients comorbid with Parkinson’s disease (n = 49) to a group of 65 non-depressed individuals (Table 1). The average ages of the mild to moderate depression group and the non depression group were 69.21 ± 3.12 years and 68.45 ± 2.34 years, respectively, with no statistically significant difference (*p* > 0.05). The onset age of PD in the comorbid group was significantly lower at 52.33 ± 3.87 years compared to 59.27 ± 3.62 years in the non-depressed group (t = 9.837, *p* < 0.001). There were no significant differences in gender distribution or BMI between the groups (*p* > 0.05). However, a higher percentage of the non-depressed group resided in urban areas compared to the mild to moderate depression group (*p* = 0.013). Other demographic characteristics, including education level, smoking history, alcohol history, hypertension, diabetes, hyperlipidemia, and marital status, did not show significant differences between the groups (*p* > 0.05).

**Table 1.** **Comparison of the demographic characteristics between the non-depressed and mild to moderate depression groups**.

Demographic characteristic		Non-depressed group (n = 65)	Mild to moderate depression group (n = 49)	t/χ^2^	*p*-value
Age (years)		68.45 ± 2.34	69.21 ± 3.12	1.487	0.140
Onset age of PD (years)		59.27 ± 3.62	52.33 ± 3.87	9.837	<0.001
Gender (male)		28/37	25/24	0.709	0.400
BMI (kg/m^2^)		24.98 ± 3.45	24.42 ± 3.21	0.884	0.379
Education level				0.902	0.342
	- Junior high school and below	34 (52.31%)	30 (61.22%)		
	- Junior high school and above	31 (47.69%)	19 (38.78%)		
Smoking history		11 (16.92%)	13 (26.53%)	1.552	0.213
Alcohol history		13 (20.00%)	15 (30.61%)	1.698	0.193
Hypertension		10 (15.38%)	12 (24.49%)	1.487	0.223
Diabetes		8 (12.31%)	13 (26.53%)	3.761	0.052
Hyperlipidemia [n (%)]		13 (20.00%)	11 (22.45%)	0.101	0.751
Residence				6.125	0.013
	Urban	35 (53.85%)	15 (30.61%)		
	Rural	30 (46.15%)	34 (69.39%)		
Marital status				1.175	0.759
	- Unmarried	5 (7.69%)	6 (12.24%)		
	- Married	31 (47.69%)	22 (44.90%)		
	- Divorced	7 (10.77%)	7 (14.29%)		
	- Widowed	22 (33.85%)	14 (28.57%)		

PD, Parkinson’s disease; BMI, Body Mass Index.

### Mood and Affective Symptoms

Patients in the mild to moderate depression group exhibited significantly higher scores on all mood and affective symptom measures compared to the non-depressed group (Table 2). Specifically, the HAM-A and Apathy Scale scores were substantially elevated in the mild to moderate depression group compared to the non-depressed group (*p* < 0.001).

**Table 2.** **Comparison of mood and affective symptoms between the non-depressed and mild to moderate depression groups**.

Mood and affective symptom	Non-depressed group (n = 65)	Mild to moderate depression group (n = 49)	t	*p*-value
HAM-A score	7.89 ± 2.56	18.45 ± 4.32	16.288	<0.001
Apathy Scale score	15.45 ± 2.98	18.34 ± 3.56	4.713	<0.001

HAM-A, Hamilton Anxiety Scale.

### Clinical Characteristics

The duration of Parkinson’s disease was significantly longer in the mild to moderate depression group (6.78 ± 2.01 years) compared to the non-depressed group (3.45 ± 1.52 years) with a t-statistic of 10.076 and a *p*-value less than 0.001 (Table 3). Additionally, a higher percentage of patients in the mild to moderate depression group were categorized in stages 1–2 of the H-Y staging compared to the non-depressed group (65.31% versus 40.00%, *p* = 0.007). However, no significant differences were observed between the groups in levodopa equivalent daily doses, motor complications, disease type, and H-Y staging categories.

**Table 3.** **Clinical characteristics of Parkinson’s disease in the non-depressed and mild to moderate depression groups**.

Parkinson’s disease characteristic		Non-depressed group (n = 65)	Mild to moderate depression group (n = 49)	t/χ^2^	*p*-value
Levodopa equivalent daily dose (mg)		600.32 ± 120.34	620.34 ± 130.54	0.848	0.398
Duration of Parkinson’s disease (years)		3.45 ± 1.52	6.78 ± 2.01	10.076	<0.001
Motor complications (yes/no)		39 (60.00%)	28 (57.14%)	0.094	0.759
Disease type					
	Tremor-dominant	20 (30.77%)	18 (36.73%)	1.230	0.541
	Stiffness-dominant	20 (30.77%)	17 (34.69%)		
	Mixed type	25 (38.46%)	14 (28.57%)		
H-Y staging				7.159	0.007
	Stages 1–2	26 (40.00%)	32 (65.31%)		
	Stages >2	39 (60.00%)	17 (34.69%)		

### Cognitive Function

The mild to moderate depression group demonstrated significantly lower scores on the MMSE (25.63 ± 2.45 versus 27.55 ± 1.78, *p* < 0.001) and on verbal fluency (10.22 ± 2.01 words/minute versus 11.76 ± 2.34 words/minute, *p* < 0.001) (Table 4). Additionally, compared to the non-depressed group, the mild to moderate depression group exhibited poorer performance on the MoCA (20.98 ± 3.12 versus 22.43 ± 3.56, *p* = 0.025) and the Trail Making Test (65.21 ± 9.67 seconds versus 60.34 ± 8.45 seconds, *p* = 0.005).

**Table 4.** **Comparison of the cognitive function between the non-depressed and mild to moderate depression groups**.

Cognitive function parameter	Non-depressed group (n = 65)	Mild to moderate depression group (n = 49)	t	*p*-value
MMSE score	27.55 ± 1.78	25.63 ± 2.45	4.847	<0.001
MoCA score	22.43 ± 3.56	20.98 ± 3.12	2.269	0.025
Verbal fluency (words/minute)	11.76 ± 2.34	10.22 ± 2.01	3.692	<0.001
Trail Making Test (seconds)	60.34 ± 8.45	65.21 ± 9.67	2.862	0.005

MMSE, Mini-Mental State Examination; MoCA, Montreal Cognitive Assessment.

### Correlation Analysis

A significant negative correlation was observed between onset age and depression (*p* < 0.001), indicating that a younger age at onset may be linked to a higher likelihood of depression (Table 5). Additionally, there was a positive correlation between Parkinson’s disease duration and depression (*p* < 0.001). Furthermore, residence exhibited a significant negative correlation with depression (*p* = 0.002), suggesting that environmental or socioeconomic factors may influence its prevalence. Additionally, cognitive function tests also showed significant negative correlations with depression. These findings highlight the complex interaction of age, disease duration, cognitive function, and environmental factors in depression among Parkinson’s disease patients.

**Table 5.** **Correlation analysis of mild to moderate depression comorbidity in Parkinson’s disease with age and various disease characteristics**.

Parameters	r	R^2^	*p*-value
Onset age of PD (years)	–0.683	0.466	<0.001
Residence	–0.302	0.091	0.002
HAM-A score	0.832	0.693	<0.001
Apathy Scale score	0.406	0.165	<0.001
Duration of Parkinson’s disease (years)	0.645	0.416	<0.001
H-Y staging	0.240	0.058	0.016
MMSE score	–0.414	0.171	<0.001
MoCA score	–0.213	0.045	0.033
Verbal fluency (words/minute)	–0.336	0.113	<0.001
Trail Making Test (seconds)	0.261	0.068	0.009

### Logistic Regression Analysis

The logistic regression analysis on Parkinson’s disease patients demonstrated several significant findings (Table 6). A younger age at onset was significantly associated with a higher likelihood of depression (*p* < 0.001), as well as a longer duration of Parkinson’s disease (*p* < 0.001). Additionally, residence showed a significant negative association with depression (*p* = 0.003), suggesting that environmental factors may play a crucial role. However, cognitive function tests indicated mixed results. These findings enhance our understanding of the factors influencing depression in Parkinson’s disease patients.

**Table 6.** **Logistic regression analysis of the association between mild to moderate depression comorbidity in Parkinson’s disease and age and underlying disease characteristics**.

Parameters	Odds ratio	95% CI	B	beta	*p*-value
Onset age of PD (years)	0.633	0.523–0.735	5.340	–0.458	<0.001
Residence	0.286	0.122–0.644	2.965	–1.253	0.003
HAM-A score	2.423	1.748–4.074	4.230	0.885	<0.001
Apathy Scale score	1.310	1.148–1.523	3.764	0.270	<0.001
Duration of Parkinson’s disease (years)	2.366	1.751–3.481	4.965	0.861	<0.001
H-Y staging	2.667	1.201–6.082	2.378	0.981	0.017
MMSE score	0.656	0.520–0.803	3.824	–0.422	<0.001
MoCA score	0.877	0.770–0.988	2.084	–0.131	0.037
Verbal fluency (words/minute)	0.724	0.586–0.874	3.196	–0.324	0.001
Trail Making Test (seconds)	1.062	1.016–1.116	2.526	0.060	0.012

CI, confidence interval.

## Discussion

The findings of this study provide valuable insights into the complex interaction of demographic, clinical, and cognitive variables in the manifestation of depressive symptoms in individuals with Parkinson’s disease. These implications can enhance the recognition and tailored management of comorbid depression, mood and affective disturbances, cognitive impairment, and disease-specific characteristics, thereby optimizing patient care and improving outcomes in PD.

The motor symptoms characteristic of Parkinson’s disease, such as tremors, rigidity, and bradykinesia, can significantly impact an individual’s quality of life and psychological well-being [20]. The challenges associated with impaired mobility and motor function can lead to social isolation, reduced physical activity, and a sense of loss of independence, all of which are known risk factors for depression [21]. These social isolations would impact individuals of different ages [22]. Our study demonstrated that patients with mild to moderate depression comorbid with PD exhibited a significantly lower onset age of PD compared to non-depressed individuals. This association highlights the potential influence of early-life experiences or genetic predisposition on the development of depressive symptoms in the context of Parkinson’s disease. It underscores the significance of considering age as a crucial factor in assessing and managing depression in PD patients. The significant association between the duration of Parkinson’s disease and the presence of mild to moderate depression further emphasizes the potential impact of disease progression on mental health outcomes. These findings corroborate existing literature [23, 24], demonstrating the complex relationship between disease progression and the increased risk of developing depressive symptoms in individuals with neurological conditions, particularly Parkinson’s disease.

There is increasing evidence of the interconnectedness between the neurobiological underpinnings of Parkinson’s disease, cognitive function, and mood disorders [25, 26]. The pathophysiological changes associated with PD, including dopaminergic system dysfunction, neuroinflammation, and neurodegeneration, can impact mood regulation and cognitive processes, contributing to the development of depression [27]. Our findings demonstrated a significant negative correlation between cognitive function scores, mood, and affective symptoms, and the presence of mild to moderate depression in Parkinson’s disease. Specifically, poorer performance on the HAM-A, Apathy Scale Score, MMSE, MoCA, verbal fluency, and Trail Making Test were observed in the mild to moderate depression group. These results underscore the intricate relationship between cognitive impairment and depressive symptoms in PD, suggesting potential overlap in the underlying neurobiological mechanisms and cognitive-emotional processing. Given the impact of cognitive impairment on overall functional status and quality of life in individuals with PD, our findings emphasize the need for approaches that address cognitive and affective domains to enhance patient outcomes.

Parkinson’s disease is known for its considerable variability in its progression, with some individuals experiencing a more aggressive course of the condition compared to others [28]. Age at onset and disease duration are closely linked to the trajectory of Parkinson’s disease, influencing the emergence of motor symptoms, cognitive decline, and non-motor manifestations [29]. By considering age-related and disease-specific variables, healthcare providers can stratify patients based on their unique disease trajectories, enabling tailored interventions and closer monitoring of those at higher risk for developing comorbid depression. The logistic regression analysis revealed that a younger age at onset and a longer duration of Parkinson’s disease were significantly associated with a higher likelihood of comorbid mild to moderate depression. These findings provide valuable prognostic insights and underscore the importance of considering age-related and disease-specific variables in risk stratification and individualized management of depression in PD patients. Additionally, the non-significant association between gender and depression prevalence highlights the need for further exploration of potential gender-specific risk factors and tailored interventions to address gender disparities in mental health outcomes in Parkinson’s disease.

Notably, our study identified a significant negative correlation between residence and depression, suggesting potential environmental or socioeconomic influences on depression prevalence in PD. Specifically, the mild to moderate depression group showed a higher proportion of the rural population and a lower proportion of the urban residents compared to the non-depressed group. This difference may be attributed to significant disparities in habits and customs between rural and urban settings. Rural patients may have greater societal and familial influences and pressures. These findings emphasize the significance of broader social determinants of health and highlight the need to consider environmental factors, including urban-rural disparities, in the assessment and management of comorbid depression in individuals with Parkinson’s disease. Addressing environmental and social aspects, alongside clinical and cognitive variables, is essential in adopting a holistic and patient-centered approach to care for individuals with PD.

Our study has certain limitations that must be acknowledged. As a retrospective case-control study, it provides insights into the associations between demographic, clinical, and cognitive variables and the occurrence of mild to moderate depression in individuals with PD. However, it cannot establish causality or long-term trajectories. Future longitudinal studies are warranted to further elucidate the temporal relationships between the identified variables and depression in the context of PD. Moreover, our study specifically focused on patients aged 65 years or older, and therefore, our findings may not be fully generalized to younger populations with PD. Additionally, our study primarily relied on self-reported and clinician-rated assessment scales, including objective measures, such as neuroimaging and biomarker data, that could provide a more comprehensive understanding of the underlying mechanisms of depression in PD.

## Conclusion

In conclusion, our study contributes to the growing body of evidence that highlights the critical role of demographic, clinical, and cognitive factors in shaping the complex landscape of depression in Parkinson’s disease. A comprehensive understanding of these factors promises to inform tailored interventions, optimize patient care, and improve outcomes in individuals living with Parkinson’s disease and comorbid depression.

## Availability of Data and Materials

Data to support the findings of this study are available on reasonable request from the corresponding author.
